# Establishment of Systems to Enable Isolation of Porcine Monoclonal Antibodies Broadly Neutralizing the Porcine Reproductive and Respiratory Syndrome Virus

**DOI:** 10.3389/fimmu.2019.00572

**Published:** 2019-03-27

**Authors:** David Goldeck, Dana M. Perry, Jack W. P. Hayes, Luke P. M. Johnson, Jordan E. Young, Parimal Roychoudhury, Elle L. McLuskey, Katy Moffat, Arjen Q. Bakker, Mark J. Kwakkenbos, Jean-Pierre Frossard, Raymond R. R. Rowland, Michael P. Murtaugh, Simon P. Graham

**Affiliations:** ^1^The Pirbright Institute, Pirbright, United Kingdom; ^2^School of Biosciences and Medicine, Faculty of Health and Medical Sciences, University of Surrey, Guildford, United Kingdom; ^3^School of Veterinary Science, Faculty of Health and Medical Sciences, University of Surrey, Guildford, United Kingdom; ^4^College of Veterinary Medicine, University of Minnesota, St. Paul, MN, United States; ^5^College of Veterinary Science and Animal Husbandry, Central Agricultural University, Aizawl, India; ^6^Faculty of Health and Medical Sciences, University of Surrey, Guildford, United Kingdom; ^7^AIMM Therapeutics, Amsterdam, Netherlands; ^8^Department of Virology, Animal and Plant Health Agency, Addlestone, United Kingdom; ^9^College of Veterinary Medicine, Kansas State University, Manhattan, KS, United States

**Keywords:** porcine reproductive and respiratory syndrome virus, B cell, antibody, heterologous protection, genetic programming

## Abstract

The rapid evolution of porcine reproductive and respiratory syndrome viruses (PRRSV) poses a major challenge to effective disease control since available vaccines show variable efficacy against divergent strains. Knowledge of the antigenic targets of virus-neutralizing antibodies that confer protection against heterologous PRRSV strains would be a catalyst for the development of next-generation vaccines. Key to discovering these epitopes is the isolation of neutralizing monoclonal antibodies (mAbs) from immune pigs. To address this need, we sought to establish systems to enable the isolation of PRRSV neutralizing porcine mAbs. We experimentally produced a cohort of immune pigs by sequential challenge infection with four heterologous PRRSV strains spanning PRRSV-1 subtypes and PRRSV species. Whilst priming with PRRSV-1 subtype 1 did not confer full protection against a subsequent infection with a PRRSV-1 subtype 3 strain, animals were protected against a subsequent PRRSV-2 infection. The infection protocol resulted in high serum neutralizing antibody titers against PRRSV-1 Olot/91 and significant neutralization of heterologous PRRSV-1/-2 strains. Enriched memory B cells isolated at the termination of the study were genetically programmed by transduction with a retroviral vector expressing the Bcl-6 transcription factor and the anti-apoptotic Bcl-xL protein, a technology we demonstrated efficiently converts porcine memory B cells into proliferating antibody-secreting cells. Pools of transduced memory B cells were cultured and supernatants containing PRRSV-specific antibodies identified by flow cytometric staining of infected MARC-145 cells and *in vitro* neutralization of PRRSV-1. Collectively, these data suggest that this experimental system may be further exploited to produce a panel of PRRSV-specific mAbs, which will contribute both to our understanding of the antibody response to PRRSV and allow epitopes to be resolved that may ultimately guide the design of immunogens to induce cross-protective immunity.

## Introduction

Porcine reproductive and respiratory syndrome (PRRS) is the most important infectious disease affecting the global pig industry. PRRS viruses (PRRSV) are a major threat to both animal welfare and food security, as demonstrated by the pig high fever disease outbreak that rapidly spread across Southeast Asia with devastating consequences (1). Annual losses to PRRSV in the USA and Europe are estimated to exceed US$600 million and €1.5 billion, respectively (2, 3). PRRSV exists as two genetically and antigenically distinct species, PRRSV-1 and -2, which are both rapidly evolving. The emergence of highly pathogenic strains from both species (1, 4, 5) and the failure of current live attenuated vaccines to provide broad protection against an ever-expanding diversity of viral strains pose significant challenges to effective disease control world-wide. There is therefore an urgent requirement to explore alternative approaches to vaccine development to combat PRRSV. Neutralizing antibodies (nAbs) confer protection against PRRSV (6) and recent studies have shown antibody responses can neutralize a wide diversity of PRRSV strains (7–11). An improved understanding of conserved antigenic targets of nAbs would enable the design of novel vaccines.

Identification of the epitopes recognized by broadly nAbs is an area of intense recent research in the context of a number of highly variable human viruses. Central to this are methods to generate and analyze the specificity of naturally occurring monoclonal antibodies (mAbs). Recent advances in methodologies to analyze antigen-specific B cells and their immunoglobulin genes are now providing large numbers of human mAbs for potential application in the design of novel immunogens. One approach with the potential to be applied to veterinary species, including the pig, involves the use of a retroviral vector to constitutively express the B cell lymphoma-6 (Bcl-6) transcription factor and the anti-apoptotic B cell lymphoma-extra large protein 1 (Bcl-xL) in memory B cells (12). With co-stimulation, transduced cells are converted into proliferating, antibody-secreting cells, amenable to cloning and analysis of their specificity in culture supernatants. This approach has been successfully deployed to isolate human mAbs capable of broadly neutralizing human parechovirus (13), respiratory syncytial virus (RSV) (12) and influenza A viruses (14), which are being used to support vaccine development (15). Additionally, the human RSV-specific mAb MEDI8897 (D25) is currently under clinical investigation as a potential passive RSV vaccine for infants (16). This approach has also been successfully used to immortalize B cells from rabbits, mice, rats, llamas, and non-human primates (12, 17).

The induction of nAbs recognizing the diverse array of PRRSV in the field is a clear and important goal for vaccine development research. We report here important first steps with the experimental induction of broad cross-protection and high titer PRRSV-neutralizing antibody responses in pigs and the adaptation of a technological system to enable the isolation of PRRSV-specific/neutralizing porcine mAbs.

## Materials and Methods

### Viruses

PRRSV-1 subtype 3 SU1-Bel and subtype 1 215-06 strains were propagated in porcine alveolar macrophages (5). PRRSV-2 strains KS06-72109 (18), KS62 (10), VR2332 (ATCC, USA), and the attenuated PRRSV-1 subtype 1 strains Olot/91 (19) and Porcillis (MSD Animal Health) were propagated in MARC-145 cells.

### Experimental PRRSV Infection of Pigs

Animal work was approved by The Pirbright Institute Animal Welfare and Ethics Committee and conducted in accordance with the UK Animals (Scientific Procedures) Act 1986. Ethical endpoints were in place and were not reached. A sequential heterologous infection study was carried out using six 12-weeks-old, PRRSV naïve, Large White/Landrace cross, female pigs. Animals were inoculated intranasally and intramuscularly with 10^6^ TCID_50_ of PRRSV-1 Olot/91. The pigs were then sequentially challenged at 35 day intervals by similar inoculation with 10^5^ TCID_50_ of PRRSV-1 SU1-Bel (day 35), PRRSV-1 215-06 (day 70) and PRRSV-2 KS06-72109 (day 105). Pigs were finally challenged with 10^7^ TCID_50_ PRRSV-1 Olot/91 (day 140) and the study terminated 7 days later (day 147). Clinical signs and rectal temperatures were measured daily and blood samples collected at weekly intervals. Serum samples were stored at −80°C for subsequent analysis and heparinized blood used for isolation of peripheral blood mononuclear cells (PBMCs), which were cryopreserved in 10% DMSO in FBS.

### PRRSV Detection by Quantitative RT-PCR

RNA was isolated from serum using the QIAamp Viral RNA Mini Kit (Qiagen, Crawley, UK) and PRRSV RNA measured by reverse transcription quantitative PCR (RT-qPCR; VetMAX™ PRRSV EU & NA RT-PCR kit, Thermo Fisher Scientific, Loughborough, UK). To determine the utility of this kit to detect the PRRSV challenge strains, RNA was extracted from aliquots of PRRSV-1 Olot/91, 215-06 and SU1-Bel, and PRRSV-2 KS06-72109 stocks and specific detection by the PRRSV-1 and PRRSV-2 primer/probes confirmed (data not shown).

### Detection of PRRSV-Specific and Neutralizing Antibody Responses In Serum

PRRSV nucleoprotein-specific serum antibody responses were determined by ELISA (PrioCHECK Porcine PRRSV Ab Strip Kit, Thermo Fisher Scientific). PRRSV-nAb titers were assessed by incubating serial 2-fold dilutions of heat-inactivated sera with 400 TCID_50_ of PRRSV for 1 h at 37°C. Virus-serum mixtures were added to MARC-145 cells (1.5 × 10^4^ cells/well) and after 72 h incubation, infection was assessed by immunofluorescence using a Cytation5 Cell Imaging Multi-Mode Reader (BioTek, Swindon, UK). nAb titers were calculated as log_2_ of the reciprocal serum dilution that fully neutralized viral replication in 50% of the wells (ND_50_).

### Enrichment of Porcine B Cell Populations From PBMC Using Magnetic- and Fluorescence-Activated Cell sorting

In a pilot study, B cells from uninfected pigs were enriched by magnetic-based cell sorting (MACSorting) for CD21^+^ cells. PBMC were suspended in 25 μl/10^6^ PBMC of CD21-PE mAb (Clone BB6-11C9-6, Cambridge Bioscience, Cambridge, UK). Cells were incubated at room temperature for 15 min in the dark, washed once in 2%FBS/PBS and re-suspended in 10 μl/10^7^ PBMC of anti-PE microbeads (Miltenyi Biotec, Bisley, UK). Cells were incubated, washed and re-suspended in 500 μl/10^8^ PBMC of 2%FBS/PBS with 5 mM EDTA (MACS buffer). Five hundred microliter of MACS buffer was then used to equilibrate the MACS MS column held within a MiniMACS magnet (Miltenyi Biotec). A volume containing ≤ 1 × 10^7^ labeled/2 × 10^8^ total PBMC was added to the column via a 70 μm cell strainer (Thermo Fisher Scientific), and washed three times with 500 μl MACS buffer. The column was removed from the magnet, CD21^+^ cells eluted with 3 ml 2%FBS/PBS and washed once in 2%FBS/PBS. Aliquots were taken post-labeling pre-enrichment and from the CD21^−^ and CD21^+^ fractions post-enrichment to assess CD21-PE labeling and enrichment. Enriched CD21^+^ B cells were labeled in 25 μl/10^6^ cells with Zombie Aqua Fixable Viability Dye (BioLegend, London, UK), biotinylated IgM (Fc) polyclonal Ab (pAb) (Cambridge Bioscience), FITC-conjugated IgA (Fc) pAb (Cambridge Bioscience), and FITC-conjugated IgG (Fc) pAb (Cambridge Bioscience) by incubation at room temperature in the dark for 15 min. All antibodies were titrated to optimal concentrations before use. Cells were washed and Brilliant Violet 421-conjugated streptavidin (BioLegend) was added 25 μl/10^6^ cells and incubated as above. Cells were then re-suspended in 100 μl/10^6^ cells IMDM with GlutaMAX (Fisher Scientific) supplemented with 2% FBS. After cells were distinguished from debris based on their FSC-A and SSC-A and singlets selected based on FSC-A and FSC-H, Zombie^−^CD21^+^IgM^+^IgG/IgA^−^ and Zombie^−^CD21^+^IgM^−^IgG/IgA^+^, were FACSorted using a BD FACSAria III cell sorter (BD Biosciences, Oxford, UK) into tubes pre-rinsed in FBS and containing 1 ml IMDM supplemented with 40% FBS. Memory B cells were enriched from previously cryopreserved PBMC from the PRRSV-hyperimmune pigs by staining with Zombie Near Infrared Fixable Viability Dye (BioLegend), biotinylated IgM pAb/streptavidin-Brilliant Violet 421, IgG-FITC pAb, IgA-FITC pAb, and IgLκ and IgLλ mAbs (clones clone 27.2.1 and 27.7.1, Celtic Diagnostics, Dublin, Ireland)/anti-mouse IgG1-Alexa Fluor 647 (Sigma, Poole, UK) and FACSorting of live IgL^+^IgM^−^IgG/IgA^+^ B-cells using a BD FACSAria Fusion cell sorter (BD Biosciences).

### Transduction of Enriched Memory B cells

MACSorted and FACSorted B cells were suspended in IMDM supplemented with 100 U/ml penicillin, 100 μg/ml streptomycin and 10% FBS (all Fisher Scientific; cIMDM) and plated 5 × 10^5^ cells/well in a 24-well tissue culture plate containing 50 ng/ml (final concentration) recombinant murine IL-21 Fc fusion protein (IL-21; AIMM Therapeutics) and 5 × 10^4^ L cells expressing human CD40 ligand (CD40L-L cells; AIMM Therapeutics) irradiated with 50 Gy of X-rays (RS2000 Biological Irradiator, Rad Source, Suwanee, USA). After 36 h culture at 37°C, B cells were harvested, washed in serum-free IMDM and transferred to 24-well non-tissue culture treated plates pre-coated with 30 μg/ml RetroNectin (Takara-Bio, Tokyo, Japan) and blocked with 2% bovine serum albumin. An equal volume of retroviral vector encoding human *Bcl-6*, human *Bcl-xL*, and *GFP* (12) was added, the plate centrifuged (754 × *g*, 45 min, room temperature) and incubated overnight at 37°C. B cells were transferred to 24-well tissue culture plates containing CD40L-L cells and IL-21 as described above. Transduced B cells were passaged every 3–4 days and transduced cell growth monitored by volumetric flow cytometry (MACSQuant Analyzer, Miltenyi Biotec). The retroviral vector containing Bcl-6 and Bcl-xL have been generated by a for-profit company, AIMM Therapeutics, which makes the plasmids available. Obtaining the plasmids requires an MTA (http://www.aimmtherapeutics.com/partnering/academic-collaboration/) that includes financial obligations.

### Assessment of Antibody Secretion by Bcl-6/Bcl-xL Transduced Porcine B Cells

MultiScreen-IP 96-well filter plates (Merck Millipore, Hertfordshire, UK) were prepared by prewetting with 15 μl/well of 35% ethanol, for 1 min, and rinsed three times with 150 μl PBS. 100 μl/well of porcine IgLκ and IgLλ mAbs at 10 μg/ml in sterile carbonate bicarbonate coating buffer (Sigma) was added and incubated at 4°C overnight. Coating antibody was decanted, wells washed thrice with IMDM, and the membrane was blocked by adding cIMDM and incubated for 2 h at 37°C. A log dilution series of transduced B cells (200, 20, and 2 cells) in cIMDM with IL-21 were added to triplicate wells before incubation for 20 h at 37°C. Wells were washed with water and 0.05% Tween20 (Sigma) in PBS (wash buffer). 100 μl/well of anti-pig IgG (H&L)-horseradish peroxidase conjugate (Cambridge Bioscience) diluted 1:5,000 in PBS was added and incubated (3 h, room temperature). The plate was washed five times with wash buffer before addition of AEC substrate solution (Thermo Fisher Scientific) and incubation (1 h, room temperature). Plates were washed with water and the membrane dried before spots were counted using an ELISpot reader (AID ELISpot System Classic, AID, Strassberg, Germany).

### Screening of Antibodies From Bcl-6/Bcl-xL Transduced Porcine B Cell Culture Supernatants

“Minipool” cultures of 10–50 transduced B cells were established in 96 well flat-bottom tissue culture plates supplemented by addition of 4 × 10^3^ irradiated CD40L-L cells and 50 ng/ml IL-21 twice weekly. Supernatants were harvested after 14 days culture and the antibody concentrations were assessed by IgG and IgA ELISA (Cambridge Biosciences). Supernatants were screened by intracytoplasmic staining of PRRSV-1 Olot/91-infected MARC-145 cells (20). Following secondary labeling with biotinylated IgG (H&L) pAb (Cambridge Biosciences)/streptavidin-Brilliant Violet 421 or Alexa Fluor 647-conjugated IgG mAb (Cohesion Biosciences, London, UK), cells were analyzed by flow cytometry. Supernatants which stained infected cells were subsequently retested by staining both uninfected and infected cells. Selected supernatants were also simultaneously screened for PRRSV-specific reactivity by staining a mixture of infected and uninfected cells, the latter of which had previously been labeled with 10 μM Tag-it Violet Proliferation and Cell Tracking Dye (BioLegend) (21). Supernatants were additionally screened for neutralizing activity against PRRSV-1 Olot/91 as described above.

### Data Analysis

Flow cytometry data was analyzed using FCS Express 6 (De Novo Software, Glendale, CA, USA), and GraphPad Prism 7.03 (GraphPad Software, La Jolla, CA, USA) was used for graphical and statistical analysis of data. One-way ANOVAs were conducted to compare changes in rectal temperatures, viral RNAemia, N-protein specific Ab and PRRSV-1 Olot/91 nAb titers over time, and, to compare nAb titers against different PRRSV strains. Two-way ANOVAs were conducted to compare the retroviral transduction and outgrowth of different porcine B cell populations as well as the assessment of their antibody secretion by ELISpot assay.

## Results

### Outcome of Heterologous PRRSV Challenge Infections and Kinetics of Antibody Responses

**Figure 1:**
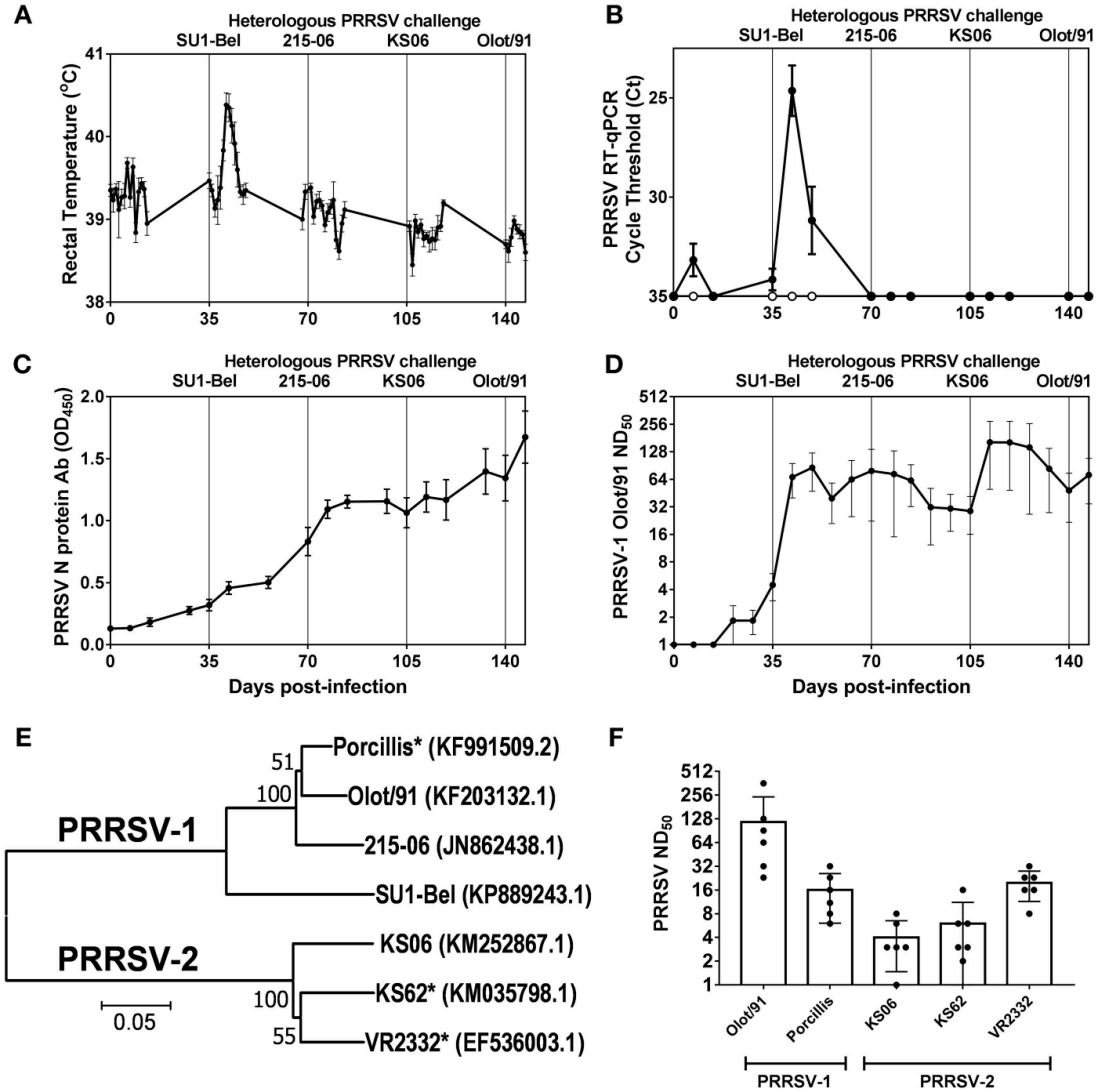
Outcome of sequential heterologous PRRSV challenge infection and kinetics of antibody responses. Six pigs were experimentally challenged by inoculation of PRRSV-1 Olot/91, SU1-Bel, 215-06, PRRSV-2 KS06-72109 (KS06), and PRRSV-1 Olot/91 at 35 day intervals. Protection against PRRSV challenges was assessed by measurement of rectal temperatures **(A)** and detection of PRRSV-1 (closed symbols) and PRRSV-2 (open symbols) RNA in serum samples **(B)**. PRRSV N protein-specific **(C)** and PRRSV-1 Olot/91-neutralizing antibodies **(D)** were measured longitudinally in serum samples. PRRSV-neutralizing serum antibodies were assessed against heterologous PRRSV-1 and−2 strains. Phylogenetic relationships between the challenge strains and heterologous strains (indicated by ^*^) were assessed based on ORF5 sequences **(E)** and neutralizing titers determined **(F)**.

Rectal temperatures and PRRSV RNA in serum were monitored longitudinally following the sequential challenges with heterologous strains spanning PRRSV-1 subtypes and PRRSV species (Figures 1A,B). Recovery from infection with the attenuated PRRSV-1 subtype 1 Olot/91 did not confer protection against challenge with the PRRSV-1 subtype 3 SU1-Bel strain. Following SU1-Bel challenge, animals experienced significantly elevated temperatures for 3 days (days 41–43; *p* < 0.05) and significant PRRSV RNAemia on days 42 and 49 (*p* < 0.01). However, following recovery from SU1-Bel infection, animals were protected against a subsequent infection with the low virulence PRRSV-1 215-06 strain and then the moderately virulent PRRSV-2 KS06-72109 strain. Assessment of PRRSV N protein-specific antibodies revealed a relatively steady increase in responses across the duration of the study. N-protein specific antibody responses were significant compared to pre-infection levels from day 42 (*p* < 0.05), however, anamnestic responses following each challenge did not achieve statistical significance (Figure 1C). PRRSV-1 Olot/91 nAbs were similarly assessed and titers were significantly elevated from day 42 (*p* < 0.05) and showed a significant increase in titers following the SU1-Bel challenge infection (*p*<0.05). There was no anamnestic response after the 216-06 challenge but titers rose again after the KS06 challenge, albeit without statistical significance (Figure 1D). To assess the breadth of the serum nAb response, the neutralization of a panel of PRRSV strains (Figure 1E) was conducted on day 147 sera (Figure 1F). nAb titers were measurable against both homologous and heterologous PRRSV-1 and−2 strains. The highest nAb titers were observed against PRRSV-1 Olot/91 which were significantly greater than those against PRRSV-1 Porcillis and PRRSV-2 strains KS06-72109 and KS62 (*p* < 0.05). The next highest nAb titers were against the heterologous PRRSV-1 Porcillis and PRRSV-2 VR2332 strains (*p* < 0.05) and lowest titers were seen against PRRSV-2 KS06-7210 and KS62.

### Assessment of the Genetic Programming of Porcine B cells

**Figure 2:**
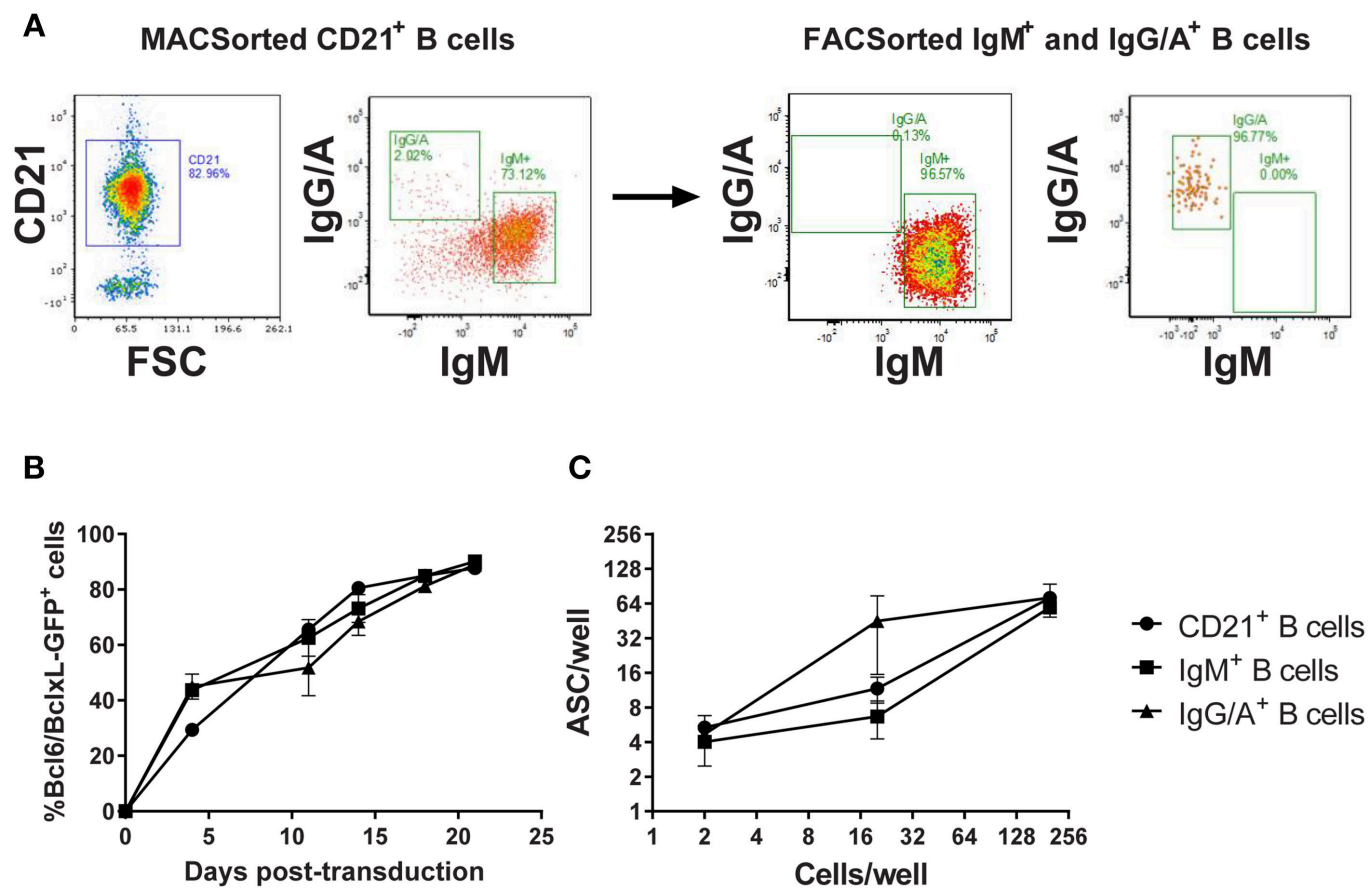
Assessment of Bcl-6/Bcl-xL retroviral transduction of porcine B cell populations. CD21^+^ B cells were enriched by MACSorting and populations of naïve/non-class switched B cells (IgM^+^) and class-switched B cells (IgG/A^+^) isolated by FACSorting **(A)**. Representative dot plots showing CD21, IgM, and IgG/A BcR staining before and after sorting are presented. CD21^+^ B cells, IgM^+^ B cells, and IgG/A^+^ B cells were transduced with the retroviral vector expressing Bcl-6/Bcl-xL/GFP and cultured with IL-21 and irradiated CD40L-L cells. Transduced cell outgrowth was tracked by assessment of % cells expressing GFP **(B)**. Active antibody secretion from transduced CD21^+^, IgM^+^, and IgG/A^+^ B cell cultures was confirmed by ELISpot assay **(C)**.

In the absence of definitive discriminatory markers, porcine memory B cells were enriched by sorting on the expression of class-switched B cell receptors (BcR). Since there was a risk that BcR labeling could induce cell-cycle arrest, a pilot experiment was conducted in which B cells were sorted based on the expression of (1) CD21, (2) IgM-BcR, or (3) IgA/IgG-BcR and transduced with the Bcl-6/Bcl-xL/GFP retroviral vector (Figure 2A). All three populations of transduced B cells proliferated with a steady increase in GFP expressing B cells between day 0 and 21 post-transduction (*p* < 0.01). Transduction efficiency was comparable (~30–50%) between the populations with all cultures approaching 100% GFP^+^ transduced cells by day 20 (Figure 2B). To assess antibody secretion, transduced B cells were incubated in ELISpot plates coated with anti-IgL and development of spots revealed the majority of B cells from transduced cultures were actively secreting antibodies (*p* < 0.01; Figure 2C).

### Screening Transduced Memory B Cells From Immune Pigs for PRRSV-Specific Antibodies

**Figure 3:**
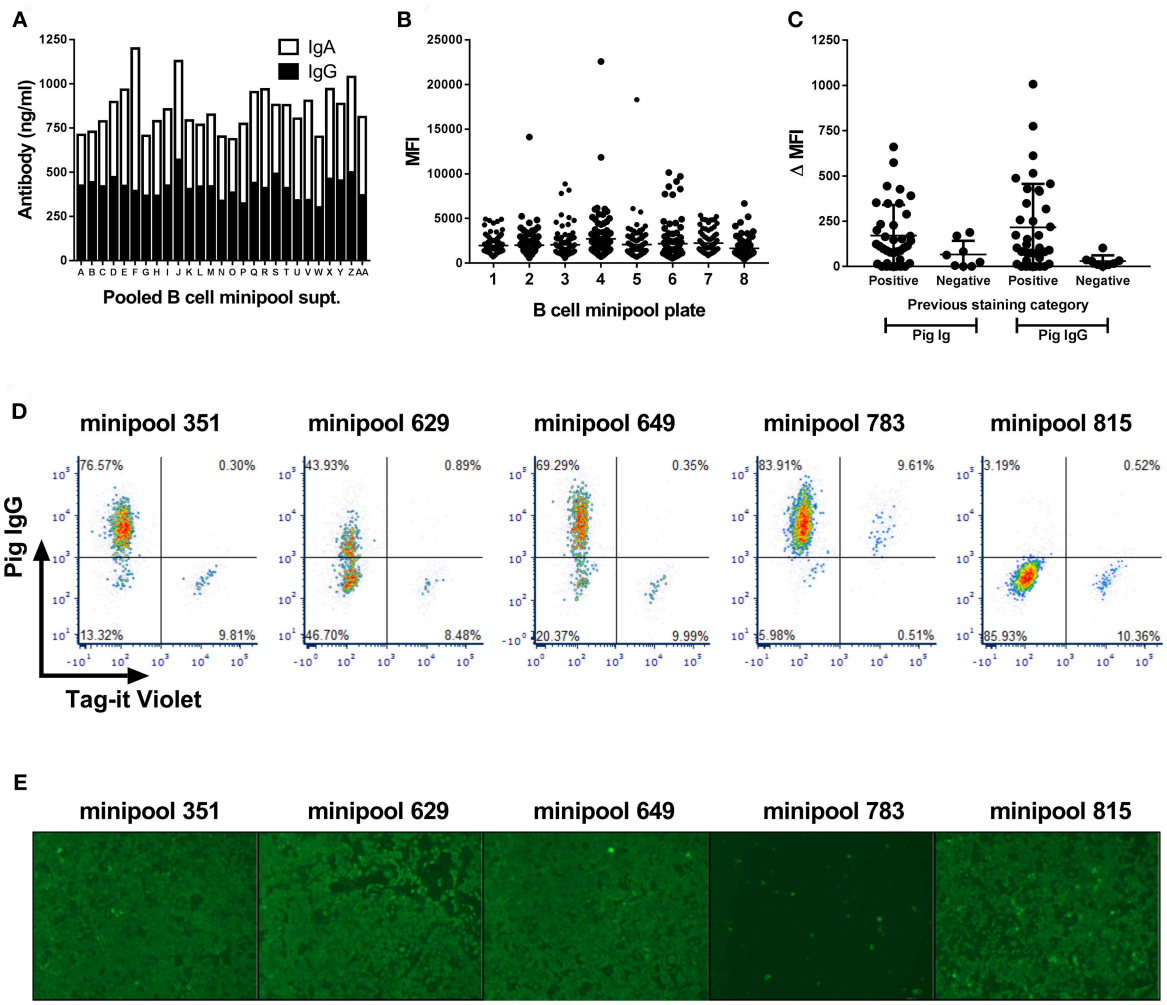
Screening of Bcl-6/Bcl-xL transduced B cell cultures for PRRSV-specific antibodies. Antibody concentrations in pooled supernatants from 768 minipool transduced B cell cultures from a single pig **(A)**. Following two week culture, supernatants were pooled and screened by IgG and IgA ELISA. Median fluorescence intensity (MFI) of PRRSV-infected MARC-145 cells stained using the 768 transduced B cell minipool culture supernatants **(B)**. Each data point represents an individual minipool B cell supernatant. Flow cytometric staining of PRRSV-1 infected and uninfected cells by selected B cell minipool supernatants **(C)**. Staining was assessed using a pan-porcine Ig secondary antibody (Pig Ig) and a porcine IgG specific secondary antibody (Pig IgG) and data presented by subtraction of the MFI of uninfected cells from the MFI of infected cells (ΔMFI). Each data point represents an individual minipool B cell supernatant and bars represent mean ± SD. Supernatants from five selected minipool B cell cultures were re-screened for PRRSV-specific antibodies by staining of mixture of PRRSV-1 infected cells and uninfected cells pre-labeled with Tag-It Violet **(D)**. Assessment of PRRSV-1 neutralization by these selected transduced B cell minipool supernatants **(E)**. Neutralization of PRRSV-1 Olot/91 was assessed by immunofluorescence staining.

Transduced B cells from the PRRSV-hyperimmune pigs seeded in minipool cultures were first screened after 2 weeks culture and shown to contain ~850 ng/ml of IgG and IgA (Figure 3A). Supernatants were next screened by intracytoplasmic staining of PRRSV-1 Olot/91 infected MARC-145 cells. Whilst the large majority of supernatants did not stain infected cells, there were a number that showed varying degrees of staining (Figure 3B), suggesting the presence of virus-specific antibodies. Forty eight pools were rescreened by parallel staining of infected and uninfected cells and a proportion of supernatants showed evidence of specific labeling of infected cells, all of which were confirmed as IgG specific (Figure 3C). With a view to improving screening throughput, a third test was performed using a pool of infected and Tag-it Violet-labeled uninfected cells allowing for correction of non-specific binding within single samples (Figure 3D). B cell minipools 351, 629, and 649 showed specific labeling of the infected population whereas minipool 783 stained both infected and uninfected cells. Finally supernatants were screened for neutralization of Olot/91 and more than 20 minipools showed evidence of virus neutralization, with three, including minipool 783 demonstrating complete neutralization (Figure 3E).

## Discussion

The aim of this study was to establish systems that would allow the isolation of PRRSV-neutralizing mAbs from pigs displaying broadly nAb responses. The first step toward this goal was the sequential infection of naïve pigs with heterologous PRRSV strains, to promote the maturation and expansion of B cells recognizing conserved neutralizing epitopes. Priming the immune system with PRRSV-1 subtype 1 Olot/91 failed to provide clinical and virological protection against the divergent subtype 3 SU1-Bel strain, which confirms earlier reports of limited cross-protection between these subtypes (22–24). However, since PRRSV-1-nAb titers were still rising at the point of SU1-Bel challenge it could be speculated that by extending the time interval beyond 35 days we could have observed greater cross-protection. However, following recovery from SU1-Bel infection, animals were protected against the subsequent challenge infections. The cross-protection against PRRSV-2 KS06 is significant since cross-protection between PRRSV species has rarely been reported (25, 26). Assessment of PRRSV-2 nAbs at the point of KS06 challenge did not show detectable titers (data not shown). However, significant nAb titers against both homologous and heterologous PRRSV-1 and−2 strains were measurable at later time-points and it may be speculated that cross-reactive B cell responses were expanded upon KS06 challenge. However, dissecting this polyclonal response is required to confirm whether such broadly reactive antibodies exist in these animals. It is well described that animals can clear PRRSV infection and be immune to reinfection in the absence of measurable nAb titers, suggesting that cross-protection may also be mediated by non-neutralizing antibody or cell-mediated responses (27–30). Non-neutralizing antibody effector functions, such as antibody dependent cell-mediated cytotoxicity, antibody-dependent complement-mediated cytotoxicity, and antibody-dependent complement-mediated virolysis, may be play roles in PRRSV immunity. Whilst the only two published studies did not provide evidence for these effector functions against PRRSV-1 (31, 32), there is still merit in evaluating non-neutralizing antibody function using the sera from these animals. Cryo-banked longitudinal PBMC samples from these animals could be used to assess T cell responses and their cross-reactivity against PRRSV-2.

The genetic programming of memory B cells is a highly effective platform technology to isolate monoclonal antibodies in a range of species (12, 17). Pilot experiments conducted with porcine B cell populations demonstrated that the system was working to expectation in this new species. The transduction efficiency for porcine B cells ranged from 30 to 50%, which is mid-range, compared to 80% for lapine B cells and 15% for camelid B cells (17). These transduction efficiencies are important to ensure a significant portion of the memory B cell repertoire is available for downstream study. Initial screening of 960 transduced B cell minipool cultures established from a single animal, provided strong evidence that PRRSV-specific B cells could be isolated using this technology. Intracytoplasmic staining of PRRSV-infected cells provided an unbiased approach to detect virus specific antibodies in transduced B cell culture supernatants. However, the screen was limited by the PRRSV strain selected. A recent study extended the fluorescent cell barcoding approach we trialed to individually labeling six populations of cells transfected with viral antigen variants (21). This allowed a quick and high-throughput approach to determine the breadth of antibody specificities. Retention of BcR expression on transduced B cells means that baiting with fluorescently labeled antigen (12) may be used to enrich for PRRSV-specific B cells, thereby significantly reducing the requirement for high-throughput screening. As proof-of-concept, we have shown that PRRSV-2 nsp7 tetramers detect rare specific B cell populations from immune pigs (33). However, the complexity of the nAb response to PRRSV and our limited understanding means that baiting with fluorescently tagged PRRSV virions (34) may provide a better approach. PRRSV-1 and−2 labeled with different dyes could allow for enrichment of mono- and pan-species specific B cells recognizing epitopes on the virion surface.

That few PRRSV-neutralizing minipool supernatants were identified is perhaps to be expected given the low serum nAb titers relative to many other virus infections. The logical next step would be to clone the B cells from these cultures to isolate mAbs which could be screened for neutralizing activity of PRRSV-1 and -2. A proteomic study revealed that PRRSV virions incorporate a variety of simian proteins after replication in MARC-145 cells (35). This raises the intriguing possibility that cross-neutralization of PRRSV strains may, in part, be mediated by antibodies recognizing simian antigens. This may also explain why minipool 783 contained antibodies that bound both infected and uninfected MARC-145 cells. To exclude this possibility, it will be important to assess neutralization in the context of both MARC-145 and macrophage infection. Sequence analysis of IGH and IGL genes would enable both their expression as recombinant mAbs and an assessment of their divergence from putative germ line predecessors (36). The longitudinal collection of PBMC from these pigs would allow a retrospective sequence based analysis of the antibody repertoire that could provide further insights into the evolution of PRRSV-nAbs (37).

There is a dire need to explore new approaches to PRRS vaccine development. We believe we have initiated an innovative approach to improve our understanding of the PRRSV-specific nAb response and isolate neutralizing mAbs. Using these neutralizing mAbs to resolve the epitopes they bind will underpin the future structure-based rational design of new PRRSV immunogens. Experimental passive immunization studies utilizing immune sera containing PRRSV-nAbs has demonstrated this to be an effective strategy (6, 11, 38). The accelerated discovery of human mAbs has meant that passive immunization strategies, for diseases where effective vaccines are lacking, is the subject of renewed interest. In addition to administration of recombinant mAbs produced in bioreactors, there are promising results being obtained by administration of vectors expressing recombinant mAbs (39–42). These vectors include mRNA, DNA and adenoviruses, which offer advantages in delivering mAbs that are difficult to produce and provide extended pharmacokinetics. Gene-based passive immunization of PRRSV-neutralizing mAbs could offer a novel, cost-effective tool in PRRS control. Our approach is also broadly applicable to the study of antibody responses to other swine pathogens. This includes bacteria (43) and as such could support vaccine development that would reduce the usage of antimicrobials in pig production. Demonstration that the genetic programming of memory B cells is effective in the pig, added to the list of species (rabbits, mice, rats, llamas, and non-human primates) previously studied (12, 17), further supports the application of this approach to other livestock or companion animal species.

## Data Availability

All datasets generated for this study are included in the manuscript and/or the supplementary files.

## Dedication

With great sorrow we mourn the passing of Dr. Michael P. Murtaugh on September 25, 2018, following complications from pancreatic cancer. Over his 33-year scientific career at the University of Minnesota, Mike made immense contributions to porcine immunology and swine diseases. He is most widely known for his significant advancements in the understanding of porcine reproductive and respiratory syndrome virus (PRRSV) evolution, pathogenesis and immunology. Mike also made extensive contributions to our fundamental understanding of the porcine immune system, antiviral immunity, molecular virology, and viral evolution, authoring over 225 peer-reviewed journal articles. His work will continue to impact the US and global swine industry for decades to come. Mike served as a mentor for over 30 Master's and PhD students, was on the editorial board of over a dozen academic journals, and successfully completed nearly 160 sponsored projects. Mike will be remembered for his dry sense of humor, caring heart, and his ability to ask difficult, yet poignant, questions that made even the sharpest grad student or post-doc sweat. He will be greatly missed.

## Author Contributions

SG, AB, MK, J-PF, RR, and MM contributed to the conception and design of the study. DG, DP, JH, LJ, JY, PR, EM, and KM performed the study and analysis. DG, DP, JH, and SG wrote the first draft of the manuscript. All authors contributed to manuscript revision, read and approved the submitted version.

### Conflict of Interest Statement

AB and MK are employed by AIMM Therapeutics. The remaining authors declare that the research was conducted in the absence of any commercial or financial relationships that could be construed as a potential conflict of interest.
